# Supplementary material for: Longitudinal Characterization of the Mumps-Specific HLA-A2 Restricted T-Cell Response after Mumps Virus Infection
Source: Vaccines (Basel). 2021 Dec 3;9(12):1431. doi: 10.3390/vaccines9121431 (PMC8707000; doi:10.3390/vaccines9121431)
Supplement: Supplementary file 1 [file vaccines-09-01431-s001.zip › vaccines-1426173-supplementary.pdf]

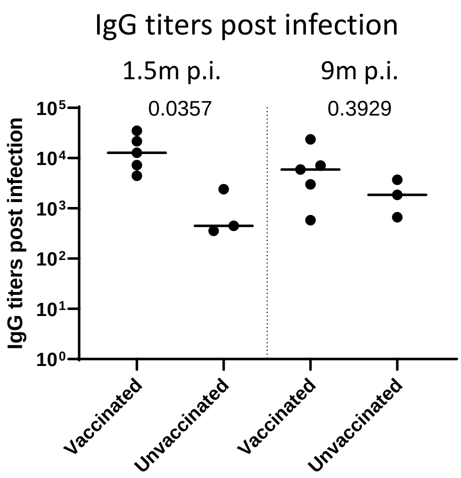

Supplementary Figure S1: Unvaccinated MuV-infected individuals have lower IgG levels 1.5 months after infection compared to vaccinated individuals.
IgG concentration (RU/ml) comparison between childhood-vaccinated (solid circles) and unvaccinated(open circles) mumps cases at 1.5 months (left) and 9 months (right) after infection. The IgG concentrations have been published before in the study of de Wit et al. 2020 [22]. Differences were calculated using the Mann-Whitney test.

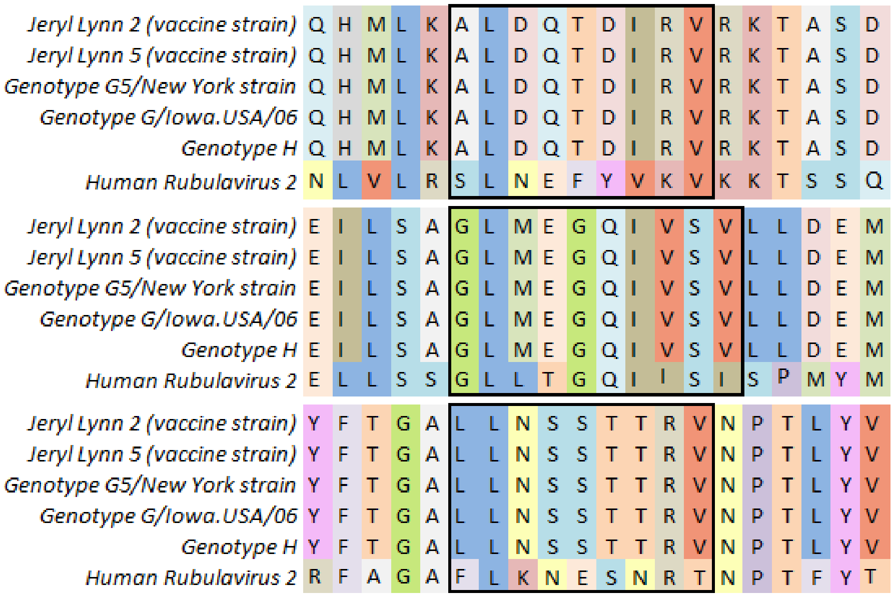

Supplementary Figure S2: The MuV-specific CD8+ epitopes are conserved within various mumps strains. Shown are the alignments of amino acid sequences of the three MuV-specific peptides (indicated by the black boxes) and their surrounding amino acids for vaccine strains Jeryl-Lynn and outbreak strains Genotype G. Human Rubulavirus 2 is used as a comparison as it belongs to the same genus as mumps virus. Positions of the epitopes: ALDQTDIRV (M-protein, residues 108-116), GLMEGQIVSV (F-protein residues 253-262), and LLDSSTTRV (HN-protein, residues 505-513).

Epitope	*MuV protein*	*Location*	*Average Tet% 1.5m*	*Average Tet% 9m*	*HLA-A2:01 Binding (%Rank)*	*Abundance (copy number/cell)*
ALDQTDIRV	M	108-116	1.17 ± 2.36	0.17 ± 0.33	1.2992	781
GLMEGQIVSV	F	253-262	0.23 ± 0.32	0.11 ± 0.23	0.0234	3
LLDSSTTRV	HN	505-513	0.85 ± 1.44	0.41 ± 0.97	0.4569	14

Supplementary Table S1: Characteristics of the MuV-specific epitopes Data about HLA-A2:01 binding was adopted from de Wit *et al.* 2020.

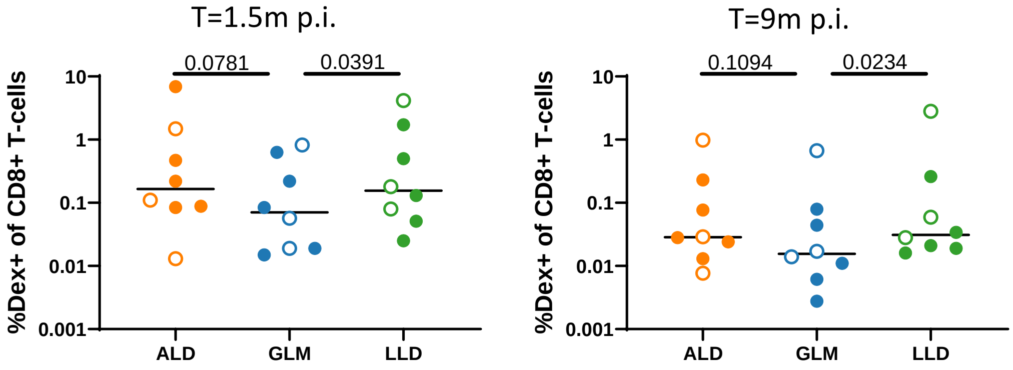

Supplementary Figure S3: Height of the CD8$^+$ T-cell frequencies differs between the MuV-specific epitopes.
Frequencies of the MuV-specific response against the three epitopes at the timepoints 1.5 months post MuV-infection (left panel) and at 9 months post MuV-infection (right panel). CD8$^+$ T cells specific for the ALD epitope are depicted in orange, for the GLM epitope in blue and for the LLD epitope in green. Solid circles indicate vaccinated individuals, whereas open circles indicated unvaccinated individuals. Wilcoxon Rank test was used to compare T-cell responses of individuals.

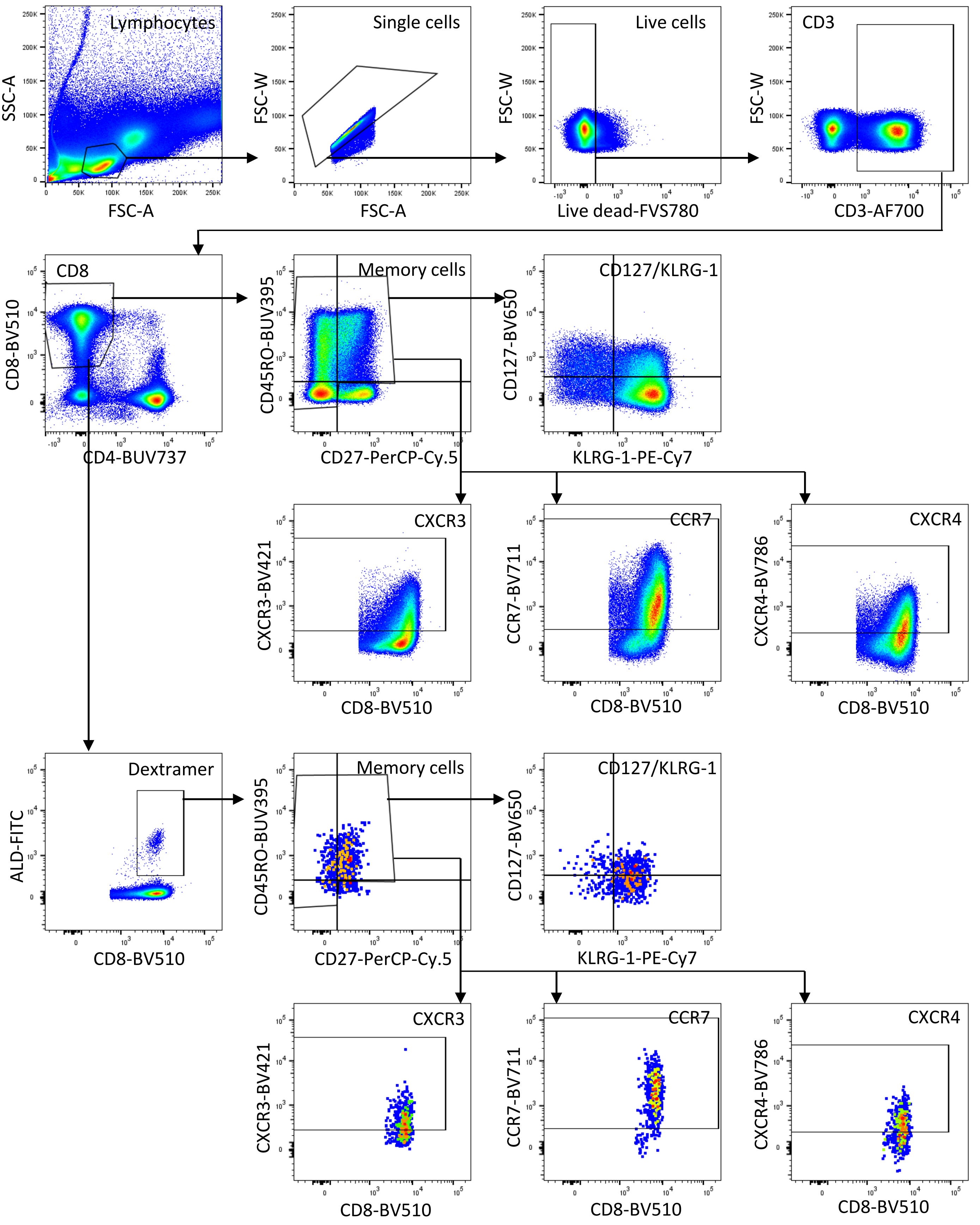

Supplementary Figure S4: Gating strategy of the phenotypical analyses of the MuV-specific response. FACS gating as used for the different cell populations in the MuV-specific (dextramer^{+}) population. Gating was based on the CD8^{+} T cell population and next applied to the MuV-specific (dextramer^{+}) populations.

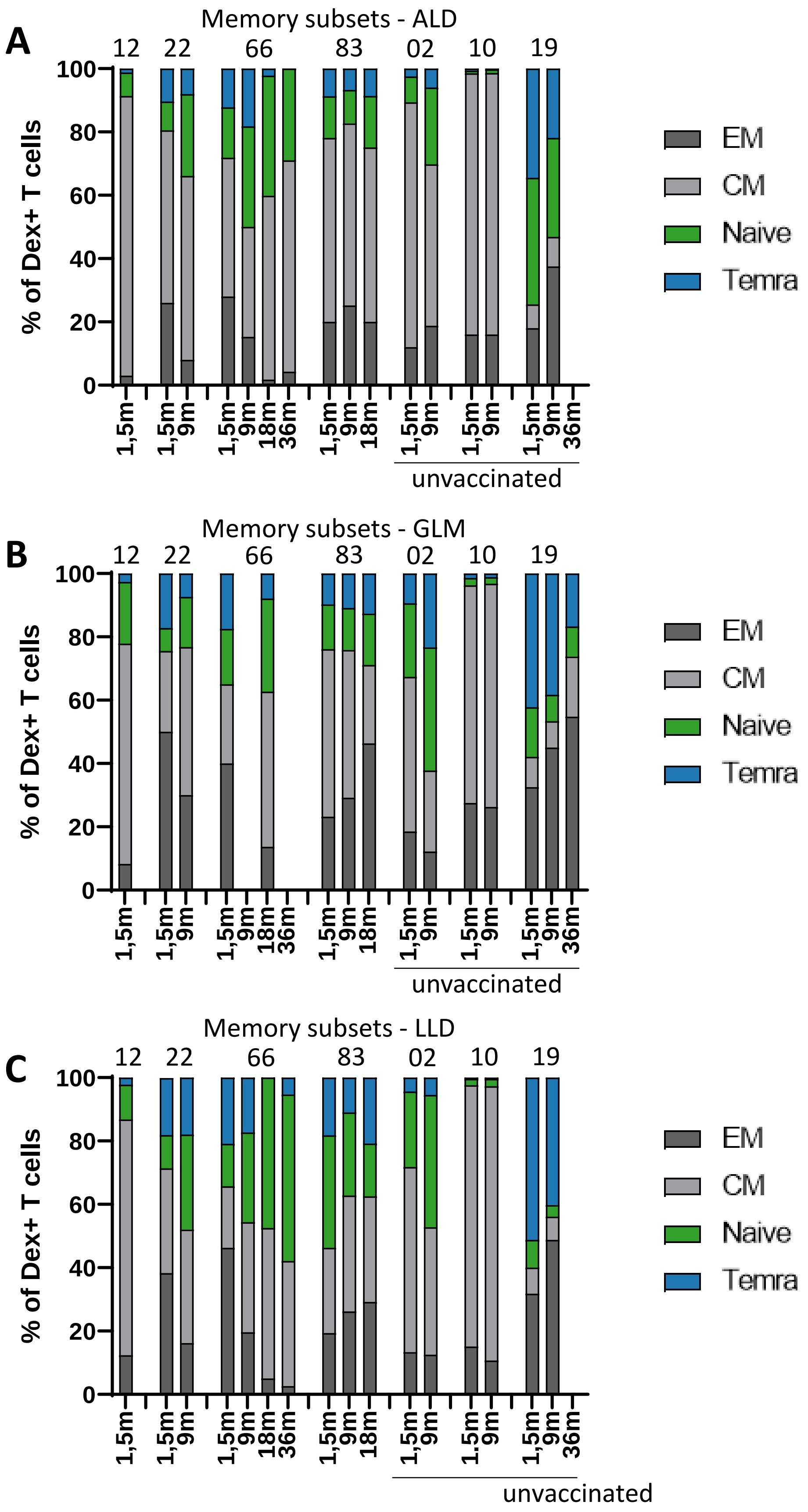

Supplementary Figure S5: Memory subsets of MuV-specific CD8$^+$ T cells show mostly patient-specific patterns instead of longitudinal dynamics.

Fraction of the memory subsets based on CD27 and CD45RO expression of the MuV-specific CD8$^+$ T cells against ALD **(A)**, GLM **(B)** and LLD **(C)** over time. The naïve T cells (CD27$^+$, CD45RO$^-$) are predicted in dark gray, Central memory (CM, CD27$^+$ CD45RO$^+$) in light gray, Effector memory (EM, CD27$^-$, CD45RO$^+$) are shown in green and the Temra cells (CD27$^-$, CD45RO$^-$) are shown in blue.

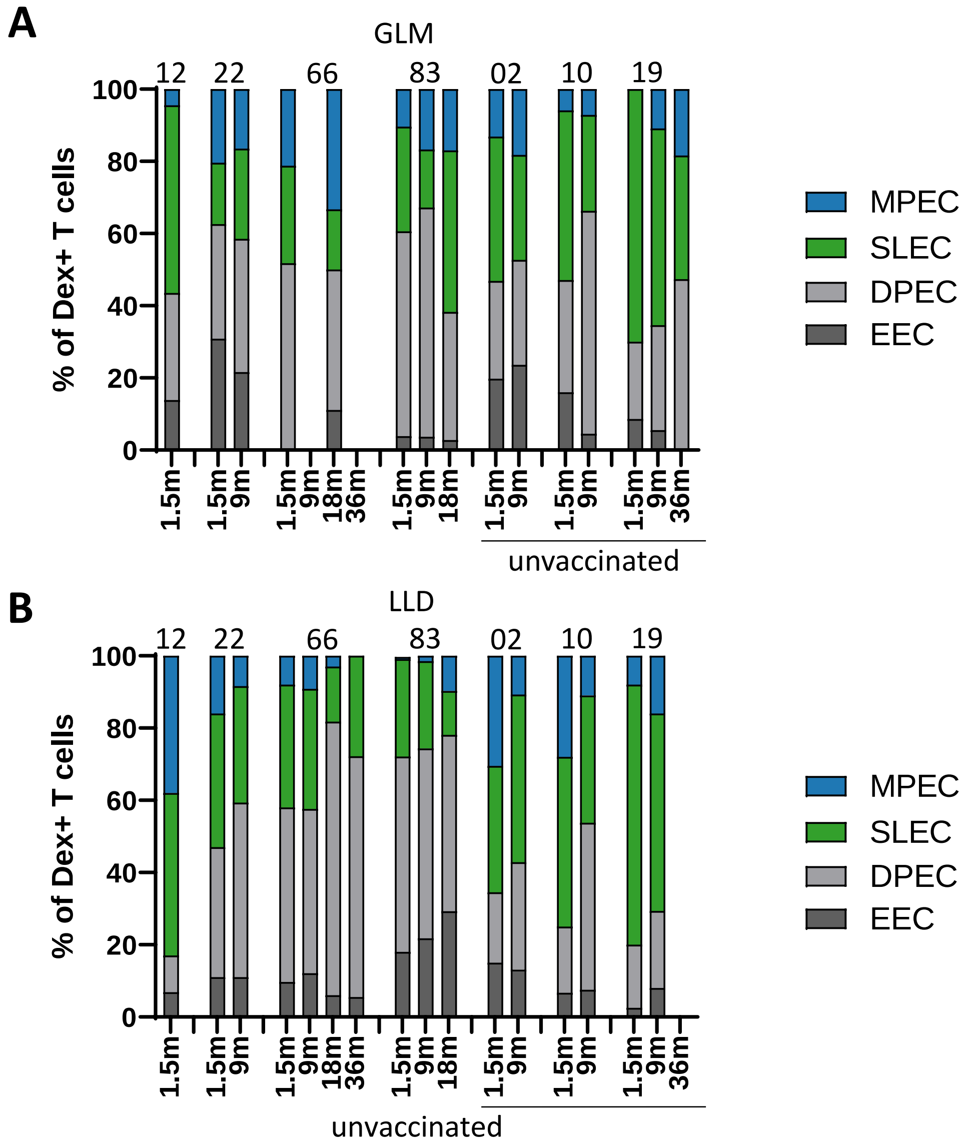

Supplementary Figure S6: MuV-specific CD8$^+$ T cells differentiate from effector cells towards memory cells over time after infection

MuV-specific CD8$^+$ T cells differentiate from effector cells towards memory cells over time after infection. Fraction of the memory subset based on CD127 Table 127. and KLRG-1 expression of the MuV-specific CD8$^+$ T cells against GLM (A) and LLD (B) over time. The memory precursors (MPEC; CD127$^+$, KLRG-1$^-$) are depicted in blue, Short lived effector cells (SLEC; CD127$^-$, KLRG-1$^+$) in green, double positive cells (DPEC; CD127$^+$, KLRG-1$^+$) are shown in light grey and the early effector cells (EEC; CD127$^-$, KLRG-1$^-$) are shown in dark grey. Donor numbers are depicted above the graphs.

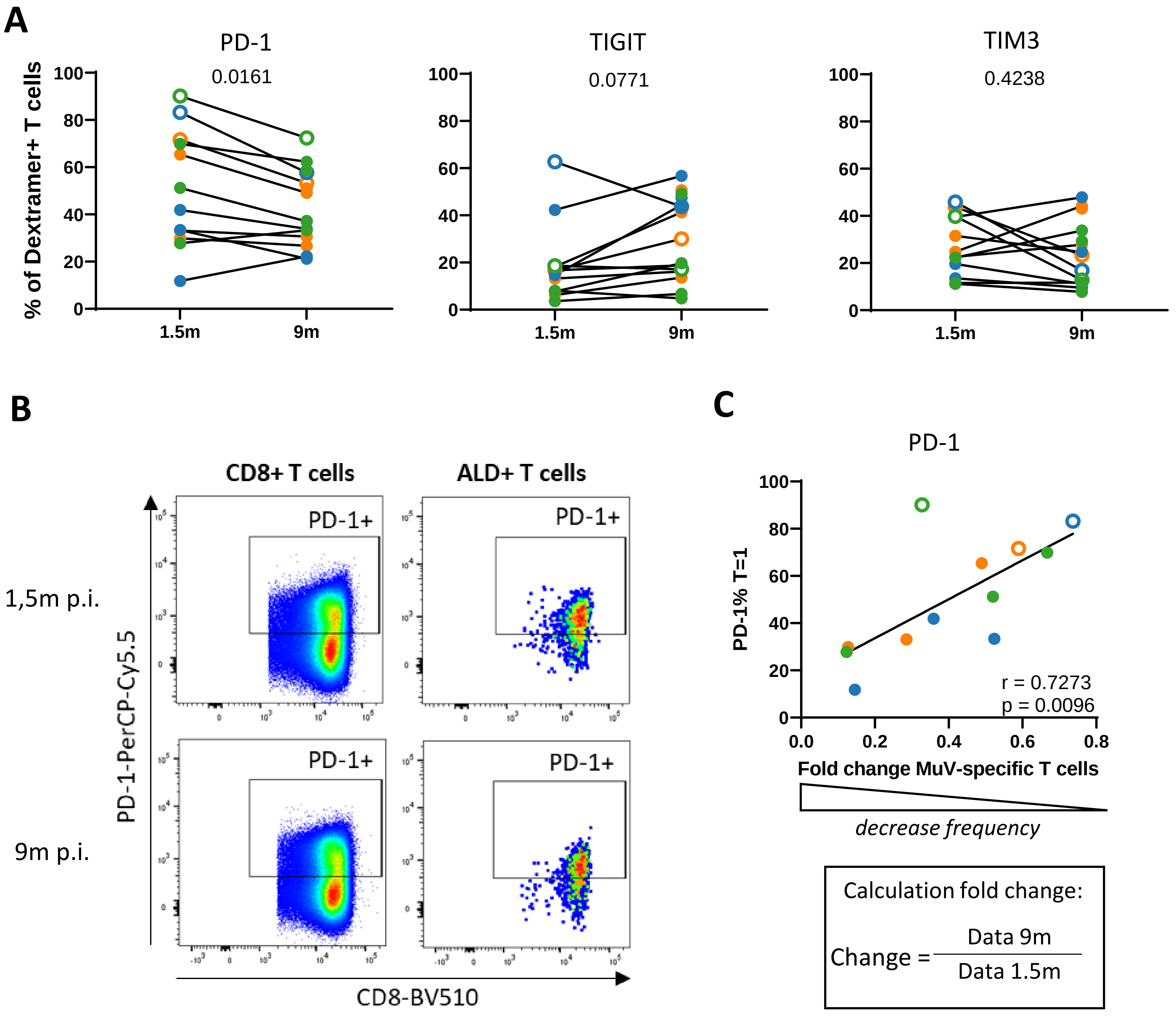

Supplementary Figure S7: PD-1$^+$ expression of the MuV-specific CD8$^+$ T cells decreases over time after infection.

(A) Fraction of de PD1$^+$, TIGIT$^+$ and TIM3$^+$ MuV-specific T cells at 1.5 months and 9 months after MuV-infection. **(B)** Gating of PD-1$^+$ T cells on both CD8$^+$ and ALD$^+$ T cells of a representative donor at both timepoint 1.5 months and 9 months after MuV-infection. **C)** Association between the expression of PD-1$^+$ MuV-specific T cells at 1.5 months after infection and the fold change in MuV-specific frequencies between 1.5 and 9 months after MuV infection.

CD8$^+$ T cells specific for the ALD epitope are depicted in orange, for the GLM epitope in blue and for the LLD epitope in green. Solid circles indicate vaccinated individuals, whereas open circles indicated unvaccinated individuals. Differences between timepoints were tested by Wilcoxon Rank test.

Fold changes were calculated by dividing the expression or frequencies found at 9 months after MuV infection by the expression or frequencies 1.5 months after MuV infection, the calculated fold changes were all below 1, indicating a decrease.

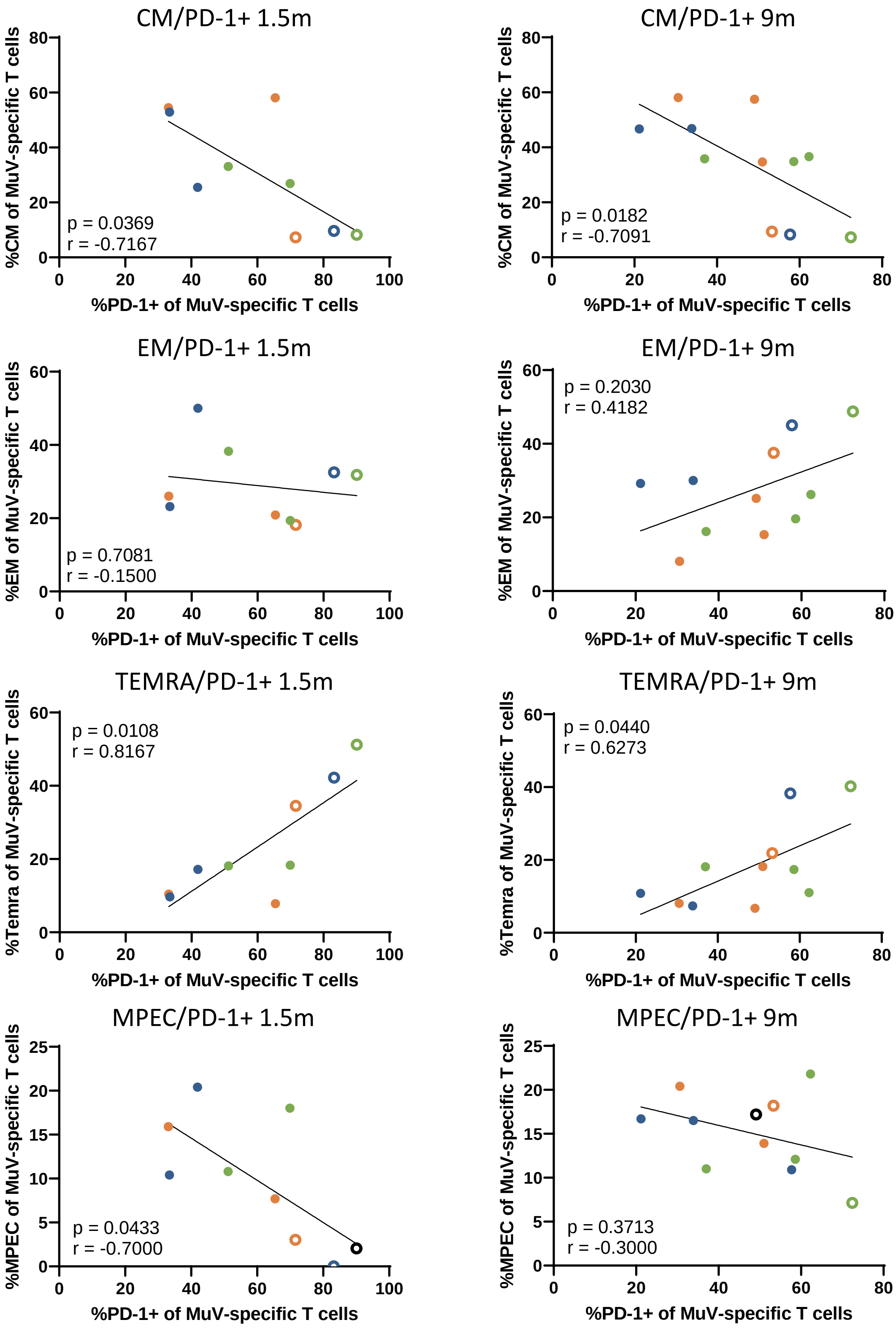

Supplementary Figure S8: PD-1 expression is associated with the memory phenotype of the MuV-specific CD8^{+} T cells

Association between the % of PD-1^{+} MuV-specific CD8+ T cells with the following subsets: CM (CD27^{+}, CD45RO^{+}), EM (CD27^{-}, CD45RO^{+}), TEMRA (CD27^{-}, CD45RO^{-}) and MPEC (KLRG-1^{-}, CD127^{+}). CD8^{+} T cells specific for the ALD epitope are depicted in orange, for the GLM epitope in blue and for the LLD epitope in green. Solid circles indicate vaccinated individuals, whereas open circles indicated unvaccinated individuals. Correlations were tested with Spearman's rank correlation coefficient.

Donor		Timepoint	# of Umi's			
83	Vac	1.5m	4	CATQEDRDRDTQYF	TRBV10-3*01	TRBJ2-3*01
			2	CASSRAGTPTDTQYF	TRBV11-3*01	TRBJ2-3*01
			1	CASSLDGGLSYNEQFF	TRBV7-2*01	TRBJ2-1*01
		9m	3	CAWSLFGHNEQFF	TRBV30*01	TRBJ2-1*01
		18m	4	CASTPGAPYGYTF	TRBV6-2*01	TRBJ1-2*01
			4	CAWSLFGHNEQFF	TRBV30*01	TRBJ2-1*01
			2	CASSRAGTPTDTQYF	TRBV11-3*01	TRBJ2-3*01
			2	CASSEFSGSPLHF	TRBV25-1*01	TRBJ1-6*01
66	Vac	1.5m	7	CASTRGGLTSSYNEQFF	TRBV12-3*01	TRBJ2-1*01
			2	CSARQGLGNEQFF	TRBV29-1*01	TRBJ2-1*01
			1	CASSLDGGVTYNEQFF	TRBV7-3*01	TRBJ2-1*01
		9m	6	CASSLDGGVTYNEQFF	TRBV7-3*01	TRBJ2-1*01
			3	CASTRGGLTSSYNEQFF	TRBV12-4*01	TRBJ2-1*01
		18m	7	CASSRGKDPQYF	TRBV7-9*01	TRBJ2-7*01
			4	CASTRGGLTSSYNEQFF	TRBV12-3*01	TRBJ2-1*01
			1	CASAGWTHYGYTF	TRBV19*01	TRBJ1-2*01
05	Vac	1.5m	7	CSARAEASGRTDTQYF	TRBV20-1*01	TRBJ2-3*01
			3	CASSLSKTPTDTQYF	TRBV11-3*01	TRBJ2-3*01
			1	CASSQEKGDVSGYTF	TRBV3-1*01	TRBJ1-2*01
		36m	9	CASSLGGQISSYNEQFF	TRBV12-4*01	TRBJ2-1*01
			4	CSARAEASGRTDTQYF	TRBV20-1*01	TRBJ2-3*01
			2	CASSLDGGLDYNEQFF	TRBV7-2*01	TRBJ2-1*01
			2	CASFLTGGRRETQYF	TRBV7-6*01	TRBJ2-5*01
			1	CASSQEAGNNQPQHF	TRBV4-3*01	TRBJ1-5*01
			1	CASSPLNGINNEQFF	TRBV7-7*01	TRBJ2-1*01
02	Unvac	1.5m	4	CATSDYGTGRAGELFF	TRBV24-1*01	TRBJ2-2*01
			3	CASSLFDLRGVANEKLFF	TRBV28*01	TRBJ1-4*01
		9m	2	CSASASGTSGSHNEQFF	TRBV20-1*01	TRBJ2-1*01
			2	CASTSTRLAANTGELFF	TRBV9*01	TRBJ2-2*01
10	Unvac	9m	21	CASRITSGGNNEQFF	TRBV27*01	TRBJ2-1*01
			9	CASSWTSTDTQYF	TRBV12-3*01	TRBJ2-3*01
12	Unvac	1.5m	11	CSAKGLAGASSSYNEQFF	TRBV20-1*01	TRBJ2-1*01
			7	CASSLTSGGNNEQFF	TRBV27*01	TRBJ2-1*01
			3	CASSLDGGVSYNEQFF	TRBV7-3*01	TRBJ2-1*01
			3	CASSGYSSGLAEETQYF	TRBV19*01	TRBJ2-5*01
			2	CATAVEMNTGELFF	TRBV15*01	TRBJ2-2*01
			2	CASSSTDYMREQYF	TRBV28*01	TRBJ2-7*01
			1	CASTTTSGLNNEQFF	TRBV27*01	TRBJ2-1*01
			1	CASSLGGLGDQPQHF	TRBV5-6*01	TRBJ1-5*01
			1	CASRLTSGGNNEQFF	TRBV27*01	TRBJ2-1*01
			1	CASSLGGQGSSYNEQFF	TRBV12-3*01	TRBJ2-1*01

Supplementary Table S2A: ALD-specific TCR sequences

Donor		Timepoint	# of Umi's			
83	Vac	1.5m	2	CSAGQGPYEQYF	TRBV29-1*01	TRBJ2-7*01
		18m	3	CAGAPDIPTDTQYF	TRBV10-3*01	TRBJ2-3*01
			1	CASSLIDFASGLNLNTGELFF	TRBV12-3*01	TRBJ2-2*01
66	Vac	1.5m	2	CAGLTSYEQYF	TRBV30*01	TRBJ2-7*01
			1	CSAYRGRPETQYF	TRBV20-1*01	TRBJ2-5*01
			1	CSAYMGRPETQYF	TRBV20-1*01	TRBJ2-5*01
		9m	3	CAISPESGRGEEANVLTF	TRBV10-3*01	TRBJ2-6*01
			2	CAISREGIGNQPQHF	TRBV10-3*01	TRBJ1-5*01
19	Vac	36m	1	CASNFQGHRAESIRILNTEAFF	TRBV12-4*01	TRBJ1-1*01
22	Vac	9m	4	CASAAENWDTQYF	TRBV7-9*01	TRBJ2-3*01
			2	CSARDLGLAGERGEYF	TRBV20-1*01	TRBJ2-3*01
			2	CSASPLLVTSPFYEQYF	TRBV20-1*01	TRBJ2-7*01
			1	CASSLEGRTVYDEQYF	TRBV5-5*01	TRBJ2-7*01
05	Vac	1.5m	1	CASSDPGVGDHYGYTF	TRBV7-9*01	TRBJ1-2*01
02	Unvac	1.5m	3	CASSLEVVFEQYF	TRBV7-9*01	TRBJ2-7*01
10	Unvac	1.5n	22	CATRGSDTGELFF	TRBV24-1*01	TRBJ2-2*01
			19	CSARDRDRAQETQYF	TRBV20-1*01	TRBJ2-5*01
			3	CASSPSGGAYTDTQYF	TRBV18*01	TRBJ2-3*01
			2	CASSPGSGNTGELFF	TRBV18*01	TRBJ2-2*01
		9m	6	CSARDRDRAQETQYF	TRBV20-1*01	TRBJ2-5*01
			5	CATRGSDTGELFF	TRBV24-1*01	TRBJ2-2*01
			2	CASSQGGQGPLNTEAFF	TRBV16*01	TRBJ1-1*01
12	Unvac	1.5m	45	CSARDRDRGSETQYF	TRBV20-1*01	TRBJ2-5*01
			7	CASSFGRTYEQYF	TRBV12-4*01	TRBJ2-7*01
			4	CASSPRDGLGNTEAFF	TRBV13*01	TRBJ1-1*01

Supplementary Table S2B: GLM-specific TCR sequences

Donor		Timepoint	# of Umi's			
66	Vac	9m	2	CSVSLGEKLFF	TRBV29-1*01	TRBJ1-4*01
			1	CASSSPGGVGDTQYF	TRBV12-3*01	TRBJ2-3*01
19	Vac	1,5m	13	CASSLGTGGYRNTEAFF	TRBV12-3*01	TRBJ1-1*01
			6	CATSREMNTEAFF	TRBV15*01	TRBJ1-1*01
			2	CATSRERNTEAFF	TRBV15*01	TRBJ1-1*01
			2	CASSTDTGSNQPQHF	TRBV19*01	TRBJ1-5*01
			1	CASSFGTGGFTNEKLFF	TRBV12-3*01	TRBJ1-4*01
		9m	2	CATSREMNTEAFF	TRBV15*01	TRBJ1-1*01
			1	CASSTDTGSNQPQHF	TRBV19*01	TRBJ1-5*01
22	Vac	1,5m	26	CSVAAEFQYEQYF	TRBV29-1*01	TRBJ2-7*01
		9m	197	CSVAAEFQYEQYF	TRBV29-1*01	TRBJ2-7*01
			29	CATSRETNTEAFF	TRBV15*01	TRBJ1-1*01
			12	CASSYEGWTEAFF	TRBV6-3*01	TRBJ1-1*01
			6	CASSQINGPEAFF	TRBV3-1*01	TRBJ1-1*01
			6	CASSQDSGLNQPQHF	TRBV4-2*01	TRBJ1-5*01
			4	CATSRRTGGINEQFF	TRBV15*01	TRBJ2-7*01
			3	CASSQDRVPNYGYTF	TRBV3-1*01	TRBJ1-2*01
			2	CASSQDALANSNEQFF	TRBV4-3*01	TRBJ2-1*01
			2	CASSQDTGSNQPQHF	TRBV4-2*01	TRBJ1-5*01
			1	CATSREINTEAFF	TRBV15*01	TRBJ1-1*01
			1	CATEQEVNTGELFF	TRBV15*01	TRBJ2-2*01
			1	CATEAERNTEAFF	TRBV15*01	TRBJ1-1*01
			1	CASRSDFNEQFF	TRBV12-4*01	TRBJ2-1*01
05	Vac	1,5m	5	CATSREMNTGELFF	TRBV15*01	TRBJ2-2*01
			3	CASSLFGEVLRWEETIYF	TRBV27*01	TRBJ1-3*01
			2	CASSQDDGSNQPQHF	TRBV4-3*01	TRBJ1-5*01
			2	CATSREWNQPQHF	TRBV15*01	TRBJ1-5*01
			2	CASSLASNFYEQYF	TRBV5-6*01	TRBJ2-7*01
			2	CASSYENYNEQFF	TRBV6-2*01	TRBJ2-1*01
			2	CASSIRSGYEQYF	TRBV19*01	TRBJ2-7*01
			1	CASSYESWTAAFF	TRBV6-3*01	TRBJ1-1*01
		9m	2	CSASWGYTEAFF	TRBV20-1*01	TRBJ1-1*01
		36m	9	CATSREAGVGETQYF	TRBV15*01	TRBJ2-5*01
			5	CASSYESWTAAFF	TRBV6-3*01	TRBJ1-1*01
			3	CASSLASNFYEQYF	TRBV5-6*01	TRBJ2-7*01
			2	CASSAWSNNQPQHF	TRBV12-4*01	TRBJ1-5*01
			1	CASSQDSGGNQPQHF	TRBV4-1*01	TRBJ1-5*01
			1	CASSVELGEQYF	TRBV9*01	TRBJ2-7*01
			1	CASSLVQKRYEQYF	TRBV12-4*01	TRBJ2-7*01

Supplementary Table S2C-1: LLD-specific TCR sequences

Donor		Timepoint	# of Umi's			
02	Unvac	1,5m	3	CATSRETNTEAFF	TRBV15*01	TRBJ1-1*01
			2	CSARDNLAASPSTDTQYF	TRBV20-1*01	TRBJ2-3*01
			2	CATSRETGSSEAFF	TRBV15*01	TRBJ1-1*01
			2	CATSREANTEAFF	TRBV15*01	TRBJ1-1*01
			1	CASSQVDYGLAKNIQYF	TRBV3-1*01	TRBJ2-4*01
10	Unvac	1,5m	60	CATSEEVNTEAFF	TRBV15*01	TRBJ1-1*01
			5	CSANPRTGEPYEQYF	TRBV20-1*01	TRBJ2-7*01
			4	CATSREAGVGETQYF	TRBV15*01	TRBJ2-5*01
			3	CASSGQGAYNSPLHF	TRBV5-1*01	TRBJ1-6*01
			3	CASSESWGQGEYNEQFF	TRBV10-1*01	TRBJ2-1*01
			3	CASSSPHGSGETQYF	TRBV12-4*01	TRBJ2-5*01
			2	CASSVELGEQYF	TRBV9*01	TRBJ2-7*01
			2	CASSDGDTQYF	TRBV6-5*01	TRBJ2-3*01
			2	CASSLVQKRYEQYF	TRBV12-4*01	TRBJ2-7*01
			2	CASGRQGNQPQHF	TRBV28*01	TRBJ1-5*01
			1	CASGDRGRQPQHF	TRBV10-2*01	TRBJ1-5*01
			1	CATSREANTEAFF	TRBV15*01	TRBJ1-1*01
			1	CATSREAGVGETL	TRBV15*01	TRBJ1-1*01
			1	CATSREAGTEAFF	TRBV15*01	TRBJ1-1*01
			1	CASRRDIGSNQPQHF	TRBV27*01	TRBJ1-5*01
			1	CARGRISGANVLTF	TRBV28*01	TRBJ2-6*01
			1	CASSISDGPNEKLFF	TRBV19*01	TRBJ1-4*01
		9m	11	CATSEEVNTEAFF	TRBV15*01	TRBJ1-1*01
			8	CATSREAGVGETQYF	TRBV15*01	TRBJ2-5*01
			1	STSRGSVAGELFF	TRBV24-1*01	TRBJ2-2*01
12	Unvac	1,5m	60	CATAVEMNTGELFF	TRBV15*01	TRBJ2-2*01
			6	CASSSNAGDYGYTF	TRBV12-4*01	TRBJ1-2*01
			3	CATSREYNTGELFF	TRBV15*01	TRBJ2-2*01
			3	CASSWESGYEQYF	TRBV6-6*01	TRBJ2-7*01
			1	CATSREYNTGELFF	TRBV15*01	TRBJ2-2*01
			1	CATSSELNTEAFF	TRBV15*01	TRBJ1-1*01
			1	CASSFKRNTEAFF	TRBV12-4*01	TRBJ1-1*01

Supplementary Table S2C-2: LLD-specific TCR sequences
